# Oral hygiene and periodontal conditions in the Chinese patients with aortic aneurysm

**DOI:** 10.1186/s12903-018-0594-3

**Published:** 2018-08-08

**Authors:** Fang Ding, Di Wu, Xiao Han, Li-Jian Cheng, Zheng Sun, Ya-Lin Lv

**Affiliations:** 1 Department of Oral Medicine, Beijing Stomatology Hospital, Capital Medical University, No.4 Tiantan Xili, Dongcheng District, Beijing, 100050 China; 2 Department of Stomatology, Beijing Anzhen Hospital, Capital Medical University, No.2 Anzhen Road, Chaoyang District, Beijing, 100029 China; 3 Beijing Anzhen Hospital, Capital Medical University, Beijing Institute of Heart, Lung and Blood Vessel Diseases, Beijing Aortic Disease Center, Beijing, China

**Keywords:** Aortic aneurysm, Cardiovascular disease, Chronic periodontitis, Clinical attachment loss

## Abstract

**Background:**

This cross-sectional study aims to evaluate the association of periodontal conditions and oral hygiene habits in the Chinese patients with an aortic aneurysm (AA).

**Methods:**

A questionnaire and periodontal examinations were carried out in the AA patients and non-AA volunteers recruited from the Center for Cardiac Surgery, Beijing Anzhen Hospital, Capital Medical University between August 2011 and June 2016. General information (e.g., height and weight), history of systemic diseases, and oral hygiene habits (e.g., brushing methods and regular oral examinations) were collected in the self-reported survey. Periodontal examinations, such as plaque index and bleeding index, were conducted in all the subjects. The correlation between periodontal indices and AA was further explored using univariate and multivariate analyses.

**Results:**

Our analyses revealed that 87.6% of AA patients have chronic periodontitis, which is significantly higher than that of the non-AA patients (55.8%). In addition, AA patients demonstrated more severe periodontal damages with 69.3% moderate and severe periodontitis, compared to only 16.0% in the non-AA group. Using AA as the dependent variable and all the potential risk factors as covariates (e.g., gender, age, smoking, obesity, diabetes, hypertension, and hyperlipidemia), a logistic regression analysis was performed to show clinical attachment loss (CAL) being an independent risk factor for AA (OR = 2.309, 95% CI: 1.623–3.284, *p* = 0.000). In comparison with the non-AA patients, more AA patients have poor oral hygiene habits and don’t have regular dental appointments for supra-gingival cleaning.

**Conclusion:**

Poor periodontal condition and dental hygiene were identified in the AA patients, suggesting that periodontitis-induced CAL may play a role in AA disease mechanisms.

## Background

Aortic aneurysm (AA), by definition, is local or diffuse aortectasia with at least 1.5 times the expected normal vascular diameter [1, 2]. AA may occur at any portion of the arterial system, including central arteries (e.g., thoracic aorta and abdominal aorta) and peripheral arteries (e.g., cerebral artery, the artery of extremities and visceral artery) [1]. AA, more commonly seen in the developed countries, is a critical condition with a male predominance and a high mortality rate once ruptures (~ 90%) [2, 3]. AA is a multifactorial disease. Risk factors for aneurysmal dilatation include trauma, infection, chronic inflammation (e.g., atherosclerosis), connective tissue disorders (e.g., Marfan syndrome) and environmental factors (e.g., smoking) [4, 5]. Clinically, AA is strongly associated with atherosclerosis, and share similar risk factors [2]. The atheromatous plaque erodes the aortic wall, destroys the arterial media and results in degeneration of elastic fibers [2, 6]. Infection and chronic inflammation may play a critical role in the initiation and development of AA [2]. Periodontitis is a chronic inflammatory disease caused by pathogenic bacteria and is correlated with cardiovascular diseases, such as atherosclerosis and AA [7]. In addition, periodontal pathogens were identified in the vascular samples from AA patients [8]. Due to the ageing of the population, changes in the lifestyles, and rise of the number of smokers, the incidence of AA in China has increased in the past few decades [9]. Thus far, epidemiological studies exploring the association between AA and periodontal diseases in the Chinese population have never been done. Our cross-sectional study aimed to investigate the periodontal conditions in AA patients, and further explore the correlation between AA and chronic periodontitis in the Chinese population.

## Methods

### Study population

Patients with aortic aneurysm (AA) were diagnosed and recruited from the Center for Cardiac Surgery, Beijing Anzhen Hospital, Capital Medical University between Jan 2012 and June 2016. This study was approved by The Institutional Review Board of Ethics Committee of Beijing Anzhen Hospital, Capital Medical University. All participants received written and oral information prior to giving written consent, and the study was performed in accordance with the Helsinki II declaration. AA was detected and diagnosed by computed tomography (CT) when the aortic diameter was 50% greater than the expected normal size. Non-AA volunteers were recruited from the health examination center as the control subjects. To diagnose chronic periodontitis, we followed the Classification of Periodontal Diseases and Conditions published by the International Workshop in 1999 [10]. Based on the level of destruction, chronic periodontitis is graded as mild (periodontal pocket depth ≤ 4 mm, clinical attachment loss (CAL) 1–2 mm, and alveolar resorption < 1/3 of root length), moderate (periodontal pocket depth ≤ 6 mm, CAL 3–5 mm, and alveolar resorption 1/3–1/2 of root length) and severe (periodontal pocket depth > 6 mm, CAL ≥ 5 mm, and alveolar resorption ≥1/2 of root length) [11]. Patients were excluded if they had periodontal treatments in the past six months and continuous antibiotics applications in the past three months, pseudo-aneurysm, and aortic dissection, or systemic diseases (e.g., malignant tumors, hepatitis and respiratory system disease).

### Questionnaire

A questionnaire was administered on all the subjects to collect general information (e.g., height, weight, education, socioeconomic status, smoking, and alcohol consumption), past medical history, history of chronic periodontitis and periodontal treatments, and dental hygiene habits. All the investigations were conducted under the clinical protocols: 2012001X, 2015011X, and 2017027X approved by the ethics committee of Beijing Anzhen Hospital, Capital Medical University. We have obtained the consent from both AA patients and non-AA volunteers before the investigation.

### Periodontal examination

The periodontal examinations were completed by the same periodontist. Plaque index (PlI), bleeding index (BI), probing depth (PD) and CAL were examined and recorded at six sites (mesial-buccal, mid-buccal, distal-buccal, mesial-lingual, mid-lingual, and distal-lingual sites) of the Ramfjord index teeth (i.e., maxillary first molar, maxillary central incisor, maxillary first premolar, mandibular first molar, mandibular central incisor, and mandibular first premolar) [10]. In addition, missing teeth (excluding third molars) were recorded.

### Statistical analysis

The data were collected and analyzed using the single-blind method by SPSS 13.0 software (SPSS Inc., Chicago, IL, USA). The measurements were demonstrated as a mean ± standard deviation and analyzed using *t*-test. Independent *t-*test was utilized for the inter-group comparison, and variance analysis was used for the comparison of multiple groups. Using AA as the dependable variable and all the potential risk factors (e.g., gender, age, and CAL) as covariates, a logistic regression analysis was performed to determine whether the periodontal infection was one of the risk factors for AA. *p* < 0.05 was considered significant.

## Results

### Patient characteristics

In total, 169 AA patients and 156 non-AA patients were included in this study. In the AA group, there were 130 male and 39 female patients, aged from 40 to 72 years (mean age 56.2 years). In the control group, 129 male and 27 female patients were recruited, aged from 40 to 73 years old (mean age 54.8 ± 5.0 years). A questionnaire was conducted to collect the background information of the patients, including gender, age, height, weight, missing teeth (Table 1) and systemic conditions (Table 2). To avoid confounding factors, we recruited patients with similar a gender ratio and age range. After matching with age and gender, the number of missing teeth was significantly higher in the AA group than in the control group. In addition, the AA group exhibited significantly higher body mass index (BMI) and the number of smokers with lower educational level (Table 1). Compared to the non-AA group, two AA patients had a family history of AA, and more AA patients had histories of coronary heart disease, hypertension, and cerebrovascular disease. In contrast, there was no statistical significance in diabetic history between two groups (Table 2).

**Table 1:** Demographical comparison between AA group and non-AA group

	Gender (M:F)	Age	BMI	Missing teeth	Smoking (Yes:No)	Education (≤ nine yrs: > nine yrs)
AA (*n* = 169)	130:39	56.2 ± 8.6	25.5 ± 4.1	1.7	99:70	93:76
Non-AA (*n* = 156)	129:27	54.8 ± 5.0	24.7 ± 3.6	0.5	74:82	35:121
*p* value	0.196	0.076	0.047*	0.000*	0.044*	0.000*

*BMI* body mass index. Independent *t*-test was performed; *: *p* < 0.05

**Table 2:** Past medical history and family history of AA group and non-AA group

	Family history of AA	Coronary heart disease	Hypertension	Cerebrovascular disease	Diabetes
AA (*n* = 169)	2	68	103	22	17
Non-AA (*n* = 156)	0	27	38	2	10
*p* value		0.000*	0.000*	0.000*	0.234

Independent *t*-test was performed; *: *p* < 0.05

### Periodontal conditions

Periodontal clinical indices, namely PLI, PD, BI, and CAL, of the Ramfjord index teeth were examined (Table 3). In addition, ten patients with chronic periodontitis were randomly selected for the self-repeatability tests of PD and CAL. The intra-group coefficients of correlation between PD and CAL reached 0.98 and 0.96, respectively. Compared to the non-AA group, the periodontal clinical indices were significantly higher in the AA group, suggestive of the association between chronic periodontitis and AA (Table 3).

**Table 3:** Comparison of periodontal clinical indices between AA group and non-AA group

	PLI	PD (mm)	BI	CAL (mm)
AA (*n* = 169)	2.4 ± 0.6	3.55 ± 0.52	3.0 ± 0.5	3.09 ± 1.27
Non-AA (*n* = 156)	2.0 ± 0.4	2.29 ± 0.49	2.1 ± 0.7	2.25 ± 1.03
*p* value	0.000*	0.000*	0.000*	0.000*

Independent *t*-test was performed; *: *p* < 0.05

In addition, the proportion of patients with periodontitis in the AA group was higher than that in the non-AA group. In particular, 87.6% of the AA patients (148/169) developed periodontitis, while 55.8% of the non-AA subjects had periodontitis (87/156). Moreover, patients in the AA group developed more severe periodontal damages, with 69.3% exhibiting moderate and severe periodontitis. In contrast, 16.0% of the non-AA subjects had moderate and severe periodontitis (Table 4). Using AA as the dependable variable and all the potential risk factors as covariates, the logistic regression analysis revealed that CAL was an independent risk factor (OR = 2.309, 95% CI, 1.623–3.284, *p* = 0.000) for AA.

**Table 4:** Comparison of periodontal status between AA group and non-AA group

Periodontal status	AA (*n* = 169)	Non-AA (*n* = 156)	*p* value
Healthy	0 (0%)	15 (9.6%)	0.000*
Gingivitis	21 (12.4%)	54 (34.6%)	0.000*
Periodontitis	148 (87.6%)	87 (55.8%)	0.000*
• Mild	31 (18.3%)	62 (39.7%)	0.000*
• Moderate	89 (52.7%)	15 (9.6%)	0.000*
• Severe	28 (16.6%)	10 (6.4%)	0.004*

Independent *t*-test was performed; *: *p* < 0.05

### Oral hygiene habits

Oral hygiene habits were investigated with a self-reported survey, including brushing methods, brushing frequency, brushing time, brushing sites, the average lifespan of a toothbrush, flossing, supra-gingival scaling and regular dental appointments. Compared to the non-AA subjects, more AA patients demonstrated inaccurate brushing method, low brushing frequency and short brushing time without dental flossing habits. Furthermore, fewer AA patients developed a routine for supra-gingival scaling and periodic dental examinations (Table 5). In our cohort, most AA patients exhibited inaccurate oral hygiene habits and lower self-awareness for oral hygiene maintenance.

**Table 5:** Comparison of oral hygiene habits between AA group and non-AA group

Oral Hygiene Habits	AA (*n* = 169)	Non-AA (*n* = 156)	*p* value
Brushing methods			
• Horizontal	63	16	0.000*
• Vertical	44	96	0.000*
• Mixed	62	44	0.103
Brushing frequency			0.001*
• < 1 time/day	82	47	
• ≥ 1 time/day	87	109	
Brushing time			
• < 1 min/time	95	52	0.000*
• 1–3 min/time	58	70	0.052
• > 3 min/time	16	34	0.002*
Brushing sites			0.012*
• Every surface	76	92	
• Only facial surfaces	93	64	
Average lifespan of a toothbrush			0.001*
• Three months	30	54	
• > Three months	139	102	
Flossing			0.026*
• Yes	6	15	
• No	163	141	
Supra-gingival scaling			0.000*
• ≥ 1 time/year	9	28	
• < 1 time/year	160	128	
Periodic dental examinations			0.003*
• Yes	15	32	
• No	154	124	

Independent *t*-test was performed; *: *p* < 0.05

## Discussion

Dental diseases are correlated with cardiovascular disorders, such as atherosclerosis-induced acute myocardial infarction [12–14]. A potential connection between periodontal diseases and cardiovascular disorders was proposed in the 1990s, followed by multiple studies supporting this hypothesis in the past two decades [15, 16]. In addition, moderate to severe periodontal diseases have been demonstrated as a significant independent risk factor for peripheral arterial diseases [17]. AA, a cardiovascular disorder, is highly associated with chronic inflammation and infections [2]. Thus far, many studies have indicated that chronic periodontitis contributes to the initiation and progression of AA [2, 7, 8]. Consistent with the previous observations, in the cohort of our current studies, 148 AA patients exhibited chronic periodontitis (87.6%), which was significantly higher than that in the non-AA group (55.8%). Compared to the non-AA subjects, AA patients exhibited poorer periodontal health, namely more missing teeth, greater PlI and CAL indices and a higher degree of periodontal damages (69.2% moderate to severe chronic periodontitis), suggesting chronic periodontitis is a risk factor for AA incidence.

Studies have revealed that periodontal pathogens, herpes simplex virus (HSV), cytomegalovirus (CMV), Epstein-Barr virus (EBV), and Chlamydia pneumoniae may be closely associated with the initiation and progression of AA [18]. Periodontal pathogens, *Aggregatibacter actinomycetemcomitans* (A. actinomycetemcomitans), were detected in 7.1% of the AA samples [19, 20]. Other bacteria species, *Porphyromonas gingivalis* (P. gingivalis), *Treponema denticola*, *Tannerella forsythia, Prevotella intermedia*, and *Campylobacter rectus*, were also identified in the AA samples [21]. Both P. gingivalis and A. actinomycetemcomitans have been successfully recovered and cultivated in the atherosclerotic tissues [22].

Periodontal pathogens were found in atherosclerotic lesions of the peripheral and central arterial systems [23]. In addition, periodontal pathogens were discovered in the AA samples, suggesting a bacterial invasion of an existing aneurysm by transient bacteremia [23]. Pathogenic microorganisms play a critical role in the initiation and progression of AA, and oral cavity and periodontal pocket may serve as the niche for these gram-negative bacteria [23]. Two hypotheses have been put forward to explain the etiopathological mechanisms of periodontitis-induced AA. The infection hypothesis proposed that microorganisms contribute to the AA pathogenesis [23]. Pathogenic bacteria from subgingival dental plaques are introduced into the bloodstream through daily activities (e.g., chewing and tooth brushing). Bacteria-induced inflammatory infiltrates then interact with the microenvironment, leading to the production of cytokines and matrix metalloproteinases (MMPs), and degeneration of collagen fibers in the arterial walls [19, 23]^.^ The second hypothesis proposed that periodontal pathogens discovered in the aneurysmal tissues were secondary to the short-term bacteremia invading the existing AA [24].

A clinical study was done to examine the prevalence of periodontitis in patients with abdominal AA or arrhythmia in the Japanese population [25]. The results demonstrated that periodontitis had greater impacts on abdominal AA progression, in comparison with other cardiovascular diseases (e.g., arrhythmia) [25]. Compared to the patients with arrhythmia, the AA patients exhibited fewer well-maintained teeth (14.6 ± 2.0 vs. 20.9 ± 0.7, *p* < 0.05) and deeper periodontal pockets (3.01 ± 0.26 vs. 2.52 ± 0.05 mm, *p* < 0.05) [25]. In addition, in another age- and gender-matched study in the Japanese patients, periodontal bacteria were detected in both abdominal AA and non-AA patients, but deeper periodontal pocket depth was identified in the AA patients [26]. Though with small sample size in these studies, the results indicated a correlation between periodontitis and AA, which is similar to our findings in the current study.

AA and coronary heart diseases share common risk factors, such as age, gender, smoking, diabetes, BMI, hypertension, and hyperlipidemia, which can also be confounding factors in the periodontal research [27, 28]. However, the pathogenesis of AA cannot be fully explained by these risk factors. In the current study, we have adjusted these risk factors in the multivariate analysis (OR = 2.309, 95% CI: 1.623–3.284, *p* = 0.000), and discovered that CAL is an independent risk factor for AA. Our results indicated the existing periodontal infections can be another risk factor in the development of AA.

Multiple studies have shown the association of periodontal conditions with AA [8, 20, 25], but this is the first study investigating the correlation between oral hygiene habits and AA in the Chinese population. Our results indicated that improper oral hygiene habits, such as inaccurate brushing method, no dental flossing, and no regular supra-gingival scaling, are correlated with the incidence of AA.

## Conclusion

Health-related knowledge and attitude to primary dental care affect the oral hygiene and oral health of an individual, and further influence the systemic health [29]. In this regard, it is essential to establish a complete oral health education program, reinforce oral hygiene maintenance and promote periodic dental care for the general population.

## Abbreviations

AA: Aortic aneurysm
CAL: Clinical attachment loss
CMV: Cytomegalovirus
CT: Computed tomography
EBV: Epstein-Barr virus
HSV: Herpes simplex virus
